# Clinical trial to assess the effect of physical exercise on endothelial function and insulin resistance in pregnant women

**DOI:** 10.1186/1745-6215-10-104

**Published:** 2009-11-17

**Authors:** Robinson Ramírez-Vélez, Ana C Aguilar, Mildrey Mosquera, Ronald G Garcia, Laura M Reyes, Patricio López-Jaramillo

**Affiliations:** 1 Nutrición Group, Universidad del Valle, Calle 4B 36-00, San Fernando, Cali, Colombia; 2 VILANO Group, Research Institute, Fundación Cardiovascular de Colombia, Street 155A N 23-58, El Bosque sector E, Floridablanca, Colombia

## Abstract

**Background:**

Preeclampsia (PE) is a common maternal disease that complicates 5 to 10% of pregnancies and remains as the major cause of maternal and neonatal mortality. Cost-effective interventions aimed at preventing the development of preeclampsia are urgently needed. However, the pathogenesis of PE is not well known. Multiple mechanisms such as oxidative stress, endothelial dysfunction and insulin resistance may contribute to its development. Regular aerobic exercise recovers endothelial function; improves insulin resistance and decreases oxidative stress. Therefore the purpose of this clinical trial is to determine the effect of regular aerobic exercise on endothelial function, on insulin resistance and on pregnancy outcome.

**Methods and design:**

64 pregnant women will be included in a blind, randomized clinical trial, and parallel assignment. The exercise group will do regular aerobic physical exercise: walking (10 minutes), aerobic exercise (30 minutes), stretching (10 minutes) and relaxation exercise (10 minutes) in three sessions per week. Control group will do the activities of daily living (bathing, dressing, eating, and walking) without counselling from a physical therapist.

**Trial registration:**

NCT00741312.

## Background

Preeclampsia is a disease of worldwide distribution, which complicates 5% to 10% of pregnancies and remains as the major cause of maternal and neonatal mortality and morbidity [1,2]. The problem is especially important in developing countries, where maternal mortality ratio is up to twenty fold higher than in developed countries [1]. In Colombia the high incidence of preeclampsia (5.1%), low birth weight (LBW; 13.2%), intrauterine growth restriction (IUGR; 7.9%) and perinatal mortality (3.68 per 1000 live births) is considered a serious public health problem that needs to be solved [3-5]. Multiple conditions such as maternal age, nulliparity, previous personal and family history of PE, poor nutrition, sedentary life styles, residence at high altitudes, and lack of adequate prenatal care have been associated with an increased risk for PE. Multiple mechanisms as oxidative stress, endothelial dysfunction, and insulin resistance, have been proposed to explain the pathogenesis of the disease [6-8]. Changes in the production and in the action of nitric oxide (NO) have been proposed as the key cause of endothelial dysfunction in preeclampsia [9]. Moreover, an increased oxidative stress has been proposed as the main responsible factor for the changes in the vascular reactivity observed during PE [9]. I It was originally proposed that the increased concentration and reactivity to vasoconstrictors observed in PE might be due to a reduction of vasodilator prostaglandins, that results in an imbalance between prostacyclin (PGI_2_) and thromboxane A_2 _(TXA_2_) [10,11]. However, this imbalance in prostaglandins has not been found in early pregnancy in women who subsequently developed PE [10].

Epidemiological evidence suggests that women who participate in programs of regular physical activity have a reduced risk of developing PE [12,13]. Several studies [14-19] have found beneficial effects of maternal physical activity during pregnancy on the delivery, the foetal growth and preterm delivery. Recently, Dempsey et al. [20] reported that physically active pregnant women experienced a 40-70% reduced risk of gestational diabetes as compared with sedentary women. Other investigations have reported that recreational physical activity during pregnancy is associated with a reduced risk of PE [21,22].

Endothelial-dependent vasodilator function can be examined non-invasively in humans by measuring brachial artery mediated dilatation [23,24] and it has been demonstrated that exercise improves flow mediated dilatation (FMD). In normal pregnancy, flow mediated dilatation increases throughout pregnancy[25,26] but the effect of exercise on FMD has not been evaluated in pregnant women.

### Rationale of the study

**Figure 1:**
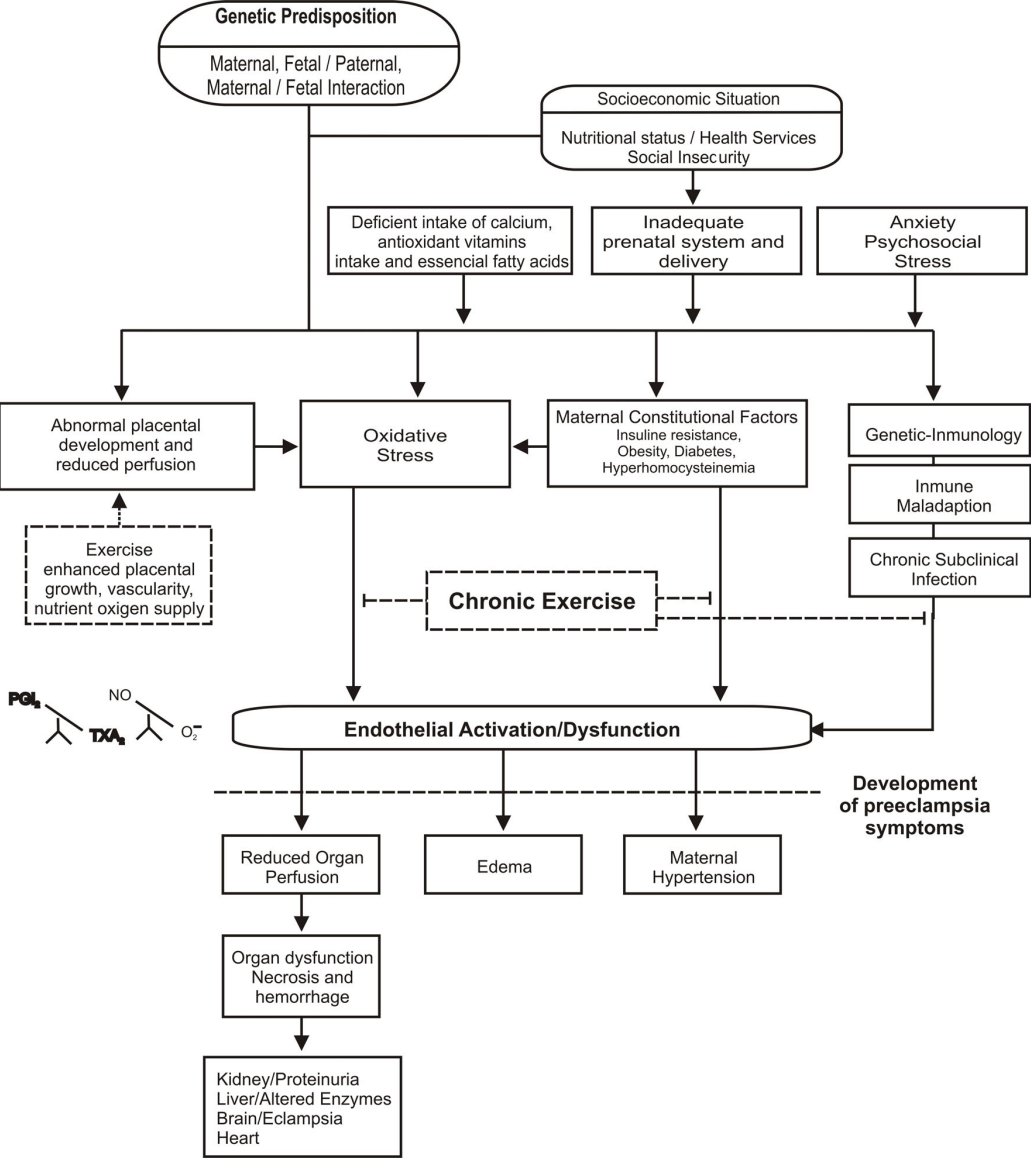
**Expected benefits of exercise during pregnancy**. Solid-line boxes represent the effects of preeclampsia and endothelial dysfunction. Dashed-line boxes represent the effects of exercise.

Evidence supports an inverse association between physical fitness and several risk factors for PE, including glucose intolerance, hypercholesterolemia, high blood pressure, obesity, and markers of systemic inflammation like C-Reactive Protein (CRP). Furthermore, physical fitness may protect against PE by intervening at three key stages of its pathogenesis: 1) Protection against abnormal placental development, 222 2) Reduction of oxidative stress, and 3) Reversal of endothelial dysfunction (Figure 1). To date, only few studies have evaluated the effects of habitual physical activity on endothelial function, and results have been controversial. In addition, no studies assessing the influence of regular aerobic exercise on endothelium-dependent brachial artery flow-mediated dilatation in pregnant women have been published. The present study will be conducted in hope of filling this gap.

## Methods/Design

### Setting

The trial will be conducted at the Universidad del Valle (UV). The Ethics Committee of Fundación Cardiovascular de Colombia (FCV) (Resolution-022/29-UV) approved the trial. All participants will provide written informed consent before entering the study.

### Design and Population

This is a single blind, randomized controlled clinical trial to evaluate the effects of a supervised aerobic exercise program on endothelium-dependent brachial artery flow-mediated dilatation in 64 nulliparous pregnant women. Interested women eligible for the present study will be invited to a pre-test including an interview (Visit A), and assessments at the Hospital Cañaveralejo and Laboratory of Biochemistry of the Universidad del Valle, Cali-Colombia. The first visit will take place between weeks 12 and 20 (Visit B), the second at week 32 (Visit C) and the last one, after delivery (Visit D).

### General objective

To determine the effect of regular aerobic exercise on endothelium-dependent brachial artery flow-mediated dilatation, insulin resistance and pregnancy outcome.

### Specific objectives

1. To determine the influence of aerobic exercise on the functional capacity (VO_2Max_), anthropometry (Fatness, body mass index [BMI]) and biochemical markers (glucose and lipid profile) in the target population.

2. To study the influence of controlled exercise on endothelial function of pregnant women.

3. To evaluate the effect of aerobic exercise on biochemical markers of insulin resistance (HOMA Index).

4. To evaluate the effect of physical exercise on maternal variables (route of delivery, duration of childbirth, postpartum complications) and the newborn (APGAR score and anthropometry).

### Outcome measurements and instruments

The primary outcome is the difference on endothelium-dependent brachial artery flow-mediated dilatation after three months of regular aerobic exercise. [Time Frame: Baseline and 32 weeks gestation]

The secondary outcome measures are High Sensitivity C-Reactive Protein, Nitrates, Nitrites, cyclic cyclic guanosine monophosphate (cGMP), blood lipid profile and glucose, HOMA index, anthropometric indicators, functional capacity (VO_2Max_), [Time Frame: Baseline and 32 weeks gestation] and maternal pregnancy outcomes (type delivery, duration of childbirth, blood pressure evolution, postpartum complications) and newborn outcomes (APGAR score and anthropometry), [Time Frame: Delivery].

### Interventions

#### Exercise Group

Regular aerobic physical exercise: Walking (10 minutes), aerobic exercise (30 minutes), stretching (10 minutes) and relaxation exercise (10 minutes) for 3 months. Exercise will be performed during 3 sessions per week. All sessions will be supervised by a physical therapist and a physical educator. The exercise-program follows the American College of obstetricians and gynecologist (ACOG) [27] and the American college of Sports Medicine (ACSM] exercise prescription [28]. Aerobic activities will be performed at moderate intensity (60-70% of maximal heart rate) measured by the 6-20 Borg's rating scale (PER). Each session will start with a 5 minutes warm up, followed by 30 minutes of aerobic activity, including a 5 minutes cool down. This is followed by 15 minutes of exercises circuit strength training of the upper limbs, lower limbs, and deep abdominal muscles stabilization. The last 5 minutesconsist of stretching and relaxation exercises.

#### Control Group

Activities of daily living: basic activities of daily living (bathing, dressing, eating, walking) without counselling from a physical therapist but including 6 sessions of prenatal behaviour control during a month [28]. *Appendix 1*.

#### Sample size

FMD is a measure of endothelial function validated in several population studies, taken as the critical variable to calculate the sample size. A sample size of 64 subjects was estimated. The sample size was calculated assuming a specific difference between the groups of 4% in the FMD after 3 months of treatment; with a maximum standard deviation of 4.5. It was accepted a type I error of 0.05, a power of 80%, and it was adjusted by 20% [29].

#### Eligibility

64 pregnant nulliparous women, healthy, 16 to 30 years old. *Appendix 2*.

#### Randomization

**Figure 2:**
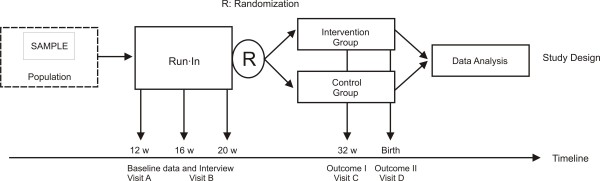
**Study design**.

Pregnant women who satisfy eligibility criteria will be randomized into one of two groups: 1) aerobic exercise group (controlled by a physical therapist and a nurse, and 2) usual prenatal control. Randomization will be performed using a variable blocking desing with a permuted block of maximum 8 subjects. Exercise will start only when each block is completed; because it consists of supervised sessions in groups of 3 to 5 women. Randomization will be administrated only by the coordinator of study, who is not involved in the intervention or in the measuring of clinical parameters. Given the nature of the interventions, it is not possible to blind the participating individuals. Instead, a research assistant at each site will control all the primary and secondary outcome assessments in order to blind to treatment assigned. While no specific qualifications or experience are required of the research assistants, training will be provided prior to the initiation of the trial about protocol and measurement procedures and methods used to maintain the blind. These procedures are also detailed in the study operations manual. Moreover, the importance of maintaining the blinding and allocation concealment will be reinforced by regularly scheduled conference calls at the sites and daily meetings with the camp investigators. Figure 2.

### Procedures

#### Enrollment

This study will be performed in pregnant women with an anticipated enrollment period of 12 months. Screening of participants will consist in the evaluation of inclusion and exclusion criteria, explanation of the study protocol, and the assessment of the willingness to participate in the study. Eligible subjects will be scheduled to the following visits:

**- Visit A: **to perform a structured validated interview (socio-demographic data, habits, medical record) and a detailed physical examination. Prenatal care visits will be continued in the primary care center assigned as part of her health insurance plan.

#### Baseline assessments and active follow-up

**- Visit B: **will include measurements of blood pressure, anthropometric parameters, FMD, functional capacity (VO_2Max_) and electrocardiogram. A fasting blood sample will be obtained to determine glucose and insulin levels, lipid profile, high sensitivity C - reactive protein (hs-CRP), nitrates, nitrites, cGMP. During this visit participants will be randomized to one of the two groups. For this purpose, a randomization system using maximum blocks of 8 will be used.

**- Visit C: **At 32 week gestation repeating measurements described above.

- **Visit D: **the following pregnancy outcomes will be recorded post partum: pregnancy duration, blood pressure evolution, characteristics of childbirth (route and duration of childbirth, postpartum hemorrhage), newborn outcomes (gestational age at birth, Apgar score, weight, height, head circumference and abdominal circumference in cm). With these measures variables will be constructed: (weight/height^3^), head circumference/birth weight and weight/height indexes [30]. In addition, the number of prenatal controls and information regarding pregnancy complications will be registered.

#### Passive follow-up

All the women included in the study will undergo a passive follow-up post delivery control 30 days after delivery (by phone).

#### Blood samples

In fasting conditions (at least 10 hours), blood samples will be withdrawn from the antecubital vein, with appropriate conditions of asepsis and antisepsis, using 3 vacutainer tubes, one dry, another with citrate, and the other containing EDTA. After 10 minutes in vertical position, all samples will be centrifuged at 3000 rpm during 15 minutes to extract the serum or plasma. Part of the samples obtained during visits B and C will be stored in Eppendorf vials at -70°C until the end of the study.

#### Physical measurements

Anthropometric will be performed in each participant at enrollment and before delivery. Standard measurements will be performed in duplicate by the same examiner on each woman. All determinations will be realized in fasting patients, wearing light clothes and without shoes, using standardized formats and methods [31].

**Weight**: will be measured with the patient standing and then registered after rounding it to the nearest 200 grams. The weight scale will be calibrated to 0 before each measurement.

**Height**: will be measured using a metric tape with the patient standing against the wall in Frankfort's position, and the value marked by a ruler placed horizontally on the head of the patient.

**Heart rate**: number of beats per minute will be measured in the radial artery.

**Blood pressure**: will be taken twice (with a difference of 5 minutes between the measurements) using a mercury sphygmomanometer on the right arm, with the patient comfortably seated, after a 5 minute rest. Systolic blood pressure (SBP) will be determined by the first audible sound (Korotkoff phase 1). Diastolic blood pressure (DBP) will be registered when the sound disappears (Korotkoff phase 5) [32]. The patient should not have smoked 30 minutes prior to the blood pressure measurement. The pneumatic arm cuff must cover 2/3 of the upper arm's length; its inferior border must be 2-3 cm over the antecubital space; the cuff will be slowly deflated. The mean blood pressure (MBP), will be calculated using the following formula [SBP+(2*DBP)]/3.

**Body composition: **% fat mass indirect (FM; kg), fat-free soft tissue mass (FFT; kg), will be measured at baseline and follow-up visits using indirect formulas [33].

**Body Mass Index (BMI)**: This index will be estimated using the weight in kilograms divided by the second power of the height expressed in meters.

##### Physical exercise protocol (Functional Capacity)

To determine the pregnancy-specific anaerobic threshold (AT), all women will perform an incremental exercise sub-maxim test of the ramp type on a mechanically braked cycloergonometer (Monarck 820K.) at a bench height to facilitate the most effective pedalling. Participants will be instructed to perform the test for as long as possible to ensure a true maximal attempt. Standard ACSM test termination criteria will be monitored and followed throughout each test. After a 3-minute warm-up at 20 W, the work rate will increase every minute by 15 wattios (W). Throughout the test the pedalling rate will be set up at 60 rpm. The pregnant women will be monitored by blood pressure and heart rate, and will be required to estimate their level of exhaustion every minute on Borg's Ratio of Perceived Exertion scale. The incremental workload will be discontinued when one of the following criteria will be met: heart rate of 160 beat per minute, or the maximum level of exhaustion on Borg's scale; another 3-minute workload will be realized at 10 W followed for cooling down before completion of the exercise test.

##### Measurement of flow-mediated dilatation of the brachial artery

Endothelium-dependent vasodilatation will be measured using the technique introduced by Celermajer et al. [23], using the guidelines reported by Corretti et al. [34]. The diameter of the brachial artery will be assessed using a high-resolution ultrasound device (Siemens SG-60, USA), equipped with a 7.5 MHz linear array transducer and an integrated electrocardiography package. The ultrasound procedures will be performed with the subject resting quietly in supine position for at least 10 minutesAll measurements will be taken at end-diastole triggered by electrocardiogram. First, the diameter of the right brachial artery will be searched in a cross-sectional view and then scanned over a longitudinal section 5 to 10 cm proximal to the right elbow. The diameter of the brachial artery will be measured from the anterior to the posterior intima/lumen interface at a fixed distance. The mean diameter will be calculated from 4 cardiac cycles. After that, a pneumatic tourniquet placed around the right forearm will be rapidly inflated to at least 50 mm Hg above the systolic blood pressure for 5 minutesA sudden release of the cuff will induce an increase in blood flow in the brachial artery located proximal to the tourniquet. During reactive hyperemia, there will be an increase in shear stress, causing endothelium-dependent vasodilatation, mainly due to endothelial release of nitric oxide [35]. This secondary dilation enhances and prolongs the reactive hyperaemic phase. FMD of the brachial artery will be measured 45-60 s after cuff release. The change in diameter caused by the increased flow will be calculated as the percentage change relative to the baseline measurement (FMD%). The dilator brachial artery response due to shear stress has been shown to have a good accuracy and reproducibility [24,36,37]. Images will be recorded on DVD player, and the measurements will be done by two observers blinded to the study and then both measurements will be averaged.

##### Biochemical markers

The routine clinical test and those of the endothelial function and inflammation markers will be processed in the Proteins, Enzymes and Biochemistry Laboratory, from the Universidad of Valle (Cali, Colombia).

**Plasma glucose levels**: Glucose levels will be measured by enzyme assay kits, using the A-15 analyser (Biosystems, España).

**Insulin**: Insulin will be determined by chemiluminescent immunometric assay (IMMULITE^® ^Automated Analyzer, Diagnostic Products Corporation, Los Angeles, USA).

**Lipid profile**: Total cholesterol, triglycerides and HDL cholesterol (HDL-C) levels will be measured by enzyme assay kits, using the A-15 analyser (Biosystems, Spain). Estimates of LDL-C concentration will be calculated using the Friedewald formula [38].

**High-sensitivity C - reactive protein (CRP): **the fasting plasma CRP concentrations will be analyzed by high sensitivity turbid meter technique using the use automatic analyzer and reagents (Biosystems, Spain) [39].

**Biomarkers of the endothelial function. Measurement of the NO in serum**: NO is rapidly converted to nitrate and nitrite. The measurement of these metabolites are used to estimate NO production The NOx (nitrate plus nitrite), concentrations in serum will be determined using a commercial kit (Nitrate/Nitrite Colorimetric Assay Kit, Cayman Chemical, USA). The within-day and between-day coefficients of variation for this assay are less than 5% at a concentration of 50 μmol/l [40]. Subjects will receive a standardized low-nitrate diet for 24 hours before testing to avoid the effect of nitrates in the diet.

**Measurement of the cGMP Level in Plasma: **blood will be collected in citrate anticoagulated tubes and immediately centrifuged. Plasma will be deproteinized by addition of 25 volumes of absolute ethanol and boiled before centrifugation. After evaporation, the sample will be dissolved in 0.05 mol/L Na-acetate buffer, pH 6.2. cGMP will be acetylated to be measured by radioimmunoassay after succinylation (Kit Colorimetric Assay. Cayman Chemical, USA) [41].

#### Nutrition

The pregnant women will answer three nutritional questions: 1) Did the participant eat meat the week before and if so, how much, 2) Did the participant eat fried food the week before and if so how often, and 3) How often did the participant eat vegetables the previous week [42].

#### Statistical Analysis

There will be an exploratory analysis which will include mean values and standard deviations of each of the variables studied to determine the balance at baseline between the groups. Normality of distribution will be checked for all variables using the Kolmogorov-Smirnov test.

The primary outcome will be the differences on endothelium-dependent brachial artery flow-mediated dilatation between the groups; and the secondary outcomes will be the differences related to anthropometric, functional and metabolic parameters, and pregnancy outcomes. The comparison between the groups will be made after treatment using the student t test for those variables with normal distribution and the non parametric Mann-Whitney test for those with non-normal distribution. Then, a linear regression analysis will be performed to determine the effect for each variable on the FMD, modeling it with Greenland parameters to determine which variable has a relationship with the model. Statistical analyses will be carried out using the SPSS software (version 14.0, SPSS, Chicago, IL). A p value < 0.05 will be considered statistically significant.

#### Study conduct and monitoring

The study will be conducted according to Good Clinical Practice and standard operating procedures. It will be monitored by the Human Rights Committee at the Universidad del Valle Coordinating Center composed of experts in physical exercise, physicians, gynecologists, nurses, physical therapists, physical educators, clinical epidemiologists and bacteriologists. Interim monitoring reports will be submitted to the experts, focusing on patient intake, adherence to protocol, baseline comparability of treatment groups, completeness of data retrieval, and adverse events. All adverse events will also be reported to the Universidad del Valle Ethics Committee. To standardize study procedures, an operations manual has been written and comprehensive training sessions will be held prior to the initiation of the trial. In addition, there will be weekly conference calls between the sites, the Coordinating Center, and the research chief to review procedures and address problems. To ensure the uniformity and quality of the interventions at each site, experts in exercise physiology will train all the therapists prior to the trial initiation and consult with them on an ongoing basis. The Coordinating Center has initiated procedures to ensure the quality and integrity of the data. These include inspections of the data forms as they are received from sites and periodic reviews of the computer data files to identify out of range values and missing data forms.

#### Ethical aspects

The clinical trial will be conducted according to the Helsinki's Declaration, the Good Clinical Practice Guidelines and the Colombian legislation (Resolution 8430/93 of the Ministry of Health). The patient will provide written informed consent in a form designed for such purpose. The information generated by the study will be confidential and strictly limited to the purposes stipulated in the protocol. The patient may refuse to continue participating in the study at any moment after providing consent. The study has been approved by both Universidad del Valle Ethics committee and that of Fundación Cardiovascular de Colombia. All assessments will be performed by trained staff. The blood samples will be collected in aseptic conditions by an expert bacteriologist. A complete description of the design and methods of this RCT, which was in accordance with CONSORT guidelines http://www.consort-statement.org was recently published [43].

#### Study timeline

**Figure 3:**
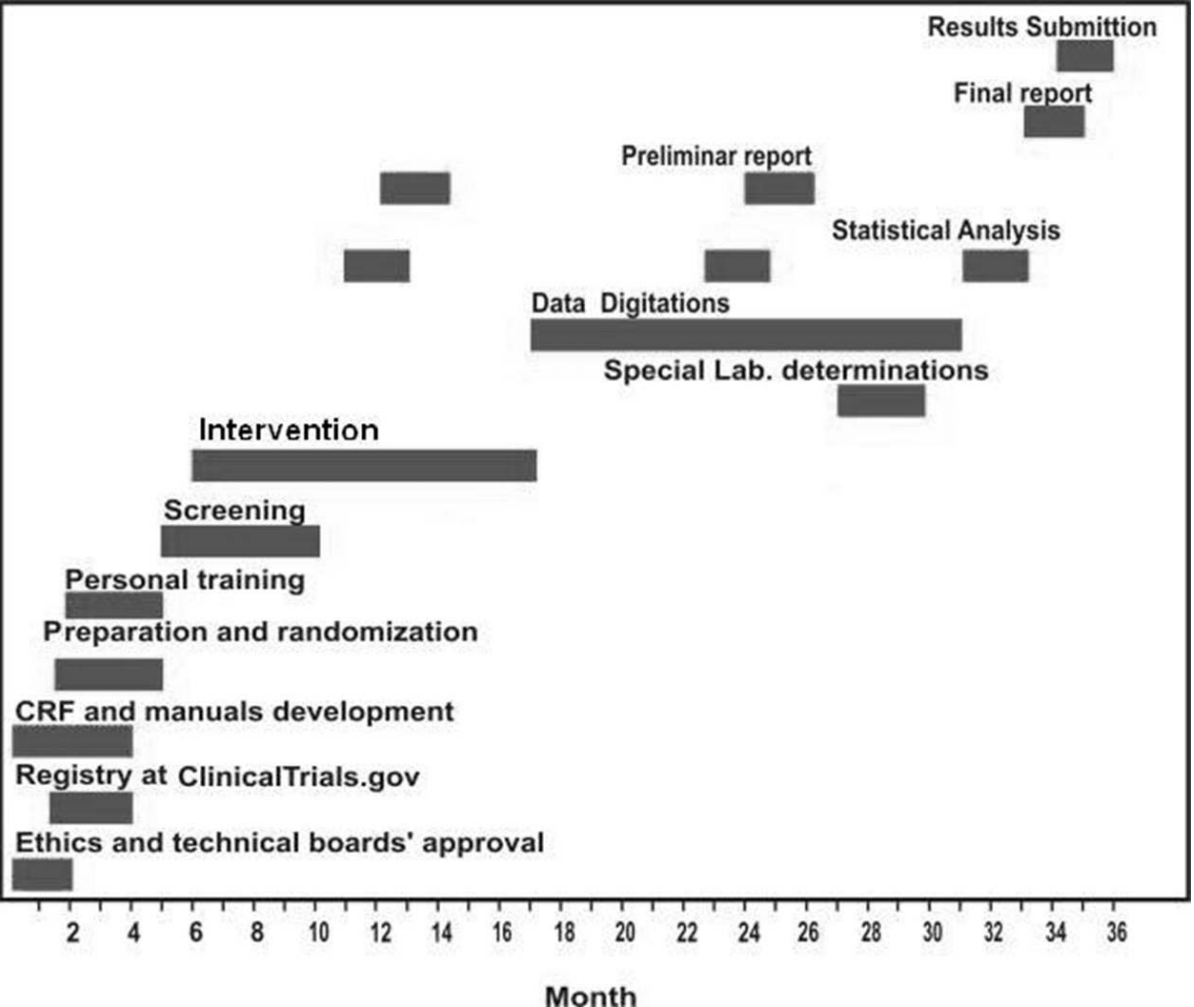
**Study Timeline**. CRF: Case report format.

The study will last 36 months. Figure 3.

## Abbreviations

PE: Preeclampsia; LBW: Low Birth Weight; IUGR: Intrauterine Growth Restriction; NO: Nitric Oxide; PGI_2_: Prostacyclin; TXA_2_: Thromboxane A_2_; FMD: Flow Mediated Dilatation; CRP: C-Reactive Protein; O_2_^-^: Superoxide; BMI: Body Mass Index; cGMP: cyclic guanosine monophosphate; ACOG: American College of obstetricians and gynaecologist; ACSM: American college of Sports Medicine; PER: Borg's rating scale; hs-CRP: High sensitivity C - reactive protein; SBP: Systolic blood pressure; DBP: Diastolic blood pressure; MBP: Mean blood pressure; W: Wattios; HDL-C: High density lipoprotein cholesterol; LDL-C: Low density lipoprotein cholesterol; NOx: Nitrate plus nitrite; CRF: Case report format.

## Competing interests

The authors declare that they have no competing interests.

## Authors' contributions

RR-V contributed in the conception and design of the manuscript. He also revised it critically and gave the final approval of the version published. ACA, MM, RG, LR and PL-J also participated in the conception and design of the manuscript, additionally they revised it critically.

## Appendices

### Appendix 1. Content of the sessions of Prenatal Behavioural Control

Session 1

Introduction

• Overview of treatment

• Instructions on how to keep activity diary; how to select activities based on PER.

Session 2

• Watch videotape on stretching and review stretching

• Supervised stretching

• Check activity diary

Session 3

• Instruct pregnant women about ways to incorporate activities into lifestyle

• Check activity diary

Session 4

• Watch videotape on relaxation exercises

• Supervised exercises

• Check activity diary

Session 5

• Instructions on maternal nutrition

• Check alimentation diary

Session 6

• Instructions on intrauterine stimulation

• Supervised activity

• Check activity diary

### Appendix 2. Selection criteria

Inclusion Criteria:

▪ Nulliparous women who have not participated in a structured exercise program during pregnancy.

▪ Living fetus at the routine ultrasound scan and a normal pregnancy.

▪ Gestational age 16 to 20 weeks.

▪ Written informed consent will be obtained from each woman prior to the inclusion in the study.

Exclusion Criteria:

▪ History of high blood pressure.

▪ Chronic medical illnesses (cancer, renal, endocrinologic, psychiatric, neurologic, infectious and cardiovascular diseases).

▪ Persistent bleeding after week 12 of gestation.

▪ Poorly controlled thyroid disease.

▪ Placenta praevia, incompetent cervix, polyhydramnios, oligohydramnios.

▪ History of miscarriage in the last twelve months.

▪ Diseases that could interfere with participation (following recommendations from ACSM 2000, ACOG 2002).
